# The causal relationship between trace element status and upper gastrointestinal ulcers: a Mendelian randomization study

**DOI:** 10.3389/fnut.2024.1443090

**Published:** 2024-10-30

**Authors:** Jianwei Liu, Gege Feng

**Affiliations:** ^1^Department of Gastroenterology, Qilu Hospital (Qingdao) of Shandong University, Qingdao, China; ^2^Department of Hematology, Qilu Hospital of Shandong University, Jinan, China

**Keywords:** causal relationship, trace element status, upper gastrointestinal ulcers, Mendelian randomization, zinc

## Abstract

**Background:**

This study aimed to investigate the bidirectional causal relationships between trace elements (such as zinc, magnesium, phosphate, and folate) and upper gastrointestinal ulcers (including gastric and duodenal ulcers). We utilized a two-sample Mendelian randomization (MR) analysis to achieve this.

**Methods:**

We conducted a two-sample MR analysis using summary-level data from genome-wide association studies (GWAS) obtained from public genomics repositories. We utilized a range of MR methods, including inverse-variance weighted (IVW), MR-Egger, and weighted median methods, and conducted a meta-analysis to synthesize results across different datasets. To ensure the robustness of our findings, we performed extensive sensitivity analyses, including pleiotropy assessment, heterogeneity tests, and leave-one-out analysis.

**Results:**

Our findings are significant, indicating a positive causal relationship between increased zinc levels and the risk of gastric ulcers. Moreover, magnesium and folate appear to offer potential protective effects against gastroduodenal ulcers (*p* < 0.05). The meta-analysis further supports the causal relationship between zinc and gastric ulcers (*p* < 0.05), confirming zinc’s significant causal impact on this condition.

**Conclusion:**

The study confirms a positive causal relationship between zinc and gastric ulcers and highlights the complexity of how trace elements regulate the progression of upper gastrointestinal ulcers. These results provide a scientific basis for dietary recommendations regarding trace element intake in clinical and public health practices. They also offer new insights into effective prevention and treatment strategies for gastric and duodenal ulcers.

## 1 Introduction

Upper gastrointestinal ulcerative diseases, specifically gastric and duodenal ulcers, have a substantial impact on the quality of life for a large global population (1, 2). These conditions not only result in significant physical discomfort but also give rise to severe complications such as bleeding, perforation, and stricture (3). The development of ulcerative diseases is closely associated with a variety of biological and environmental factors, with the most prevalent factors being persistent infection with *Helicobacter pylori* (4), and chronic use of non-steroidal anti-inflammatory drugs (NSAIDs) (5). These factors play a role in the development of ulcers by compromising the integrity of the gastric mucosal barrier, enhancing gastric acid secretion, or causing direct harm to gastric mucosal cells. In recent years, there has been a growing focus on the impact of trace elements on gastrointestinal well-being (6–9). These essential minerals, needed in small quantities, are vital for sustaining various biological and physiological processes. Both inadequate and excessive levels of trace elements can have profound implications for human health (10–12). Inadequacies may manifest in various diseases and dysfunctions, including compromised immune function, impaired growth and development, and cognitive disorders (13, 14). Conversely, overconsumption of trace elements can result in toxic reactions that impair organ function and potentially lead to organ damage (15, 16). Thus, the maintenance of a balanced intake of trace elements and a comprehensive understanding of their interactions and overall impact on human health represent crucial focal points for research in the field of nutritional science. For example, zinc is a cofactor for numerous enzymes and is involved in immune function, protein synthesis, and cell division. Its anti-inflammatory and antioxidant properties are crucial for preserving the integrity of the gastrointestinal mucosa and facilitating self-repair mechanisms. Studies have indicated that disruptions in zinc metabolism are linked to a higher prevalence of gastrointestinal disorders (17, 18). Selenium is an antioxidant that plays a critical role in cell defense. Its deficiency has been linked to gastric cancer and other gastrointestinal illnesses (19, 20). The dysregulation of iron and copper metabolism has been noted in connection with gastrointestinal disorders. Iron, necessary for oxygen transportation and cellular proliferation, can induce oxidative damage to the gastric mucosa when present in excessive amounts (21, 22). Copper—a constituent of numerous vital enzymes—plays a role in supporting the immune system and antioxidant mechanisms (23); disruptions in copper levels can similarly impact gastrointestinal well-being (24, 25).

Epidemiological research has offered initial indications of an association between deficiencies in trace element status and gastrointestinal disorders. However, the findings of these studies are frequently subject to confounding variables, complicating the establishment of causal relationships. Discrepancies in lifestyle, genetic predispositions, and other unaccounted-for environmental factors may impede the accuracy of these observations. Additionally, the majority of investigations have concentrated on individual or limited trace elements, neglecting potential interactions and intricate biological systems.

To better understand the potential causal relationship between trace element statuses and upper gastrointestinal ulcerative diseases, we used data on upper gastrointestinal ulcers (specifically gastric ulcers, duodenal ulcers, and gastroduodenal ulcers) from the Finngen database (R10 version) (https://r10.risteys.finngen.fi/), a public genomics repository, as outcomes (26), and genome-wide association study (GWAS) data pertaining to various trace element statuses from the OpenGWAS database (a public genomics repository)^1^ as exposures (27, 28). The exposure-related datasets included calcium.ionized (GCST90012623), iron (GCST90012683), magnesium (GCST90012696), phosphate (GCST90012717), folate (GCST90012742), vitamin D (VD, GCST90012771), cobalamin (vitamin B_12_, VB_12_, GCST90012772), retinol (GCST90012773), pyridoxine (GCST90012774), copper (ieu-a-1073), selenium (ieu-a-1077), and zinc (ieu-a-1079). Utilizing Mendelian randomization (MR) to investigate the causal associations between trace element statuses and upper gastrointestinal ulcerative diseases, as well as employing reverse MR analysis to examine the impact of upper gastrointestinal ulcers on trace element statuses, this bidirectional approach offers novel insights and methodological advancements for comprehending their interplay (29, 30). Our findings not only elucidate the complex biological roles of trace elements in upper gastrointestinal ulcerative diseases but also lay the groundwork for targeted prevention and treatment strategies. By integrating genetic, epidemiological, and bioinformatic approaches, we have deepened our understanding of the pathogenesis of these conditions, paving the way for more accurate prediction, prevention, and treatment methods. This multidisciplinary approach enables the development of more effective, comprehensive disease management protocols, offering refined guidance for the precise clinical management of upper gastrointestinal ulcers. Ultimately, this research advances our ability to address these diseases with increased precision and efficacy.

## 2 Materials and methods

### 2.1 Data sources

In this study, exposure data were obtained from the openGWAS database, including various trace element statuses, including calcium.ionized (GCST90012623), iron (GCST90012683), magnesium (GCST90012696), phosphate (GCST90012717), folate (GCST90012742), VD (GCST90012771), cobalamin (VB_12_, GCST90012772), retinol (GCST90012773), pyridoxine (GCST90012774), copper (ieu-a-1073), selenium (ieu-a-1077), and zinc (ieu-a-1079). Outcome data for upper gastrointestinal ulcers were selected from the Finngen database (R10 version, gastric ulcers: 6,459 cases, 350,064 controls; duodenal ulcers: 3,795 cases, 350,064 controls; gastroduodenal ulcers: 10,021 cases, 350,064 controls) for initial analysis, and from the openGWAS database (gastric ulcers: 6,293 cases, 467,985 controls; duodenal ulcers: 1,291 cases, 359,903 controls; and gastroduodenal ulcers: 3,467 cases, 357,727 controls) for validation analysis. Two-sample MR analysis was conducted separately on the initial and validation datasets. There was a requirement that datasets originate from European populations, with the largest sample size or case count being preferred when the same phenotype was found in multiple datasets. Reporting follows the strengthening of the reporting of observational studies in epidemiology using the Mendelian randomization (STROBE-MR) statement (31).

### 2.2 Instrumental variables selection

#### 2.2.1 Identification of exposure-related IVs

The “TwoSampleMR” R package was used to identify single nucleotide polymorphisms (SNPs) closely associated with exposures to serve as IVs (32). The parameters for selecting IVs were set as follows: *p* = 5e-06, *r*^2^ = 0.001, kb = 10,000 (33). The selection of IVs adhered to three assumptions of MR analysis: the relevance assumption, asserting a strong association between the SNP and the exposure; the independence assumption, indicating that the SNP is independent of confounding factors; and the exclusion restriction assumption, where the SNP affects the outcome solely through the exposure (34). To ensure the validity of these IVs, preliminary quality checks focused on verifying their sample sizes and effect allele frequencies (EAFs). Missing sample size data were supplemented from respective database information while missing EAF data were calculated using data from the “1,000 Genomes Project” (35).

#### 2.2.2 Exclusion of confounding factors

To eliminate potential confounding IVs, the LDtrait Tool from the “LDlink” database^2^ was employed (36, 37). IVs associated with phenotypes linked to gastric or duodenal diseases were identified as potential confounders and excluded in the current study. Additionally, SNPs are known to be closely associated with risk factors for gastroduodenal diseases, such as NSAIDs drug use, high stress (38), and “*H. pylori*” infection (4), were also defined as confounding SNPs and excluded from the analysis. Following these steps, the retrieved IVs underwent *F*-statistic calculations to ensure all IVs included in the study had an *F*-value above 10 to verify their suitability and accuracy (39–41).

#### 2.2.3 Identification of outcome-related IVs

Based on the types of IVs closely related to the exposure, the “TwoSampleMR” R package was used to extract IVs associated with the study outcomes, setting parameters as follows: rsq = 0.8, maf_threshold = 0.3 (32). The exposure and outcome-related IVs were then harmonized, ensuring their effect and reference alleles matched, and the “MR-PRESSO” R package was utilized to identify and remove outlier IVs (42). Following these data-cleaning steps, the final list of IVs for MR analysis was obtained. Additionally, we performed the same quality control and IV screening in the reverse analysis.

### 2.3 MR analysis

The MR analysis was primarily conducted using the “TwoSampleMR” R package, where the initial analysis involved different types of trace element statuses as exposures with upper gastrointestinal ulcers as outcomes (32). Validation analysis was performed using data from different databases to further evaluate the causal effects of various trace element statuses on upper gastrointestinal ulcers. Both initial and validation analyses employed the same MR methods, including reverse MR analysis. Inverse variance weighted (IVW) was the primary method, supplemented by the MR Egger (43), weighted median (44), simple mode (45), and weighted mode (46–48) methods. To identify significance, the IVW method must have a *p*-value less than 0.05, and the *p* values from the five methods must be in agreement (49). The “TwoSampleMR” package also provided odds ratios (ORs) and their 95% confidence intervals (CI) to aid in assessing the MR analysis’s causal effects.

### 2.4 Sensitivity analysis

Sensitivity analysis was conducted to determine whether the included IVs were pleiotropic or heterogeneous (50). Heterogeneity was tested using the *Q-*test when more than two SNPs were available in an analysis direction, and heterogeneity was indicated if the value of the P*_Q test_* was less than 0.05 (51, 52). In such cases, the “random effects model-IVW” method was employed to reassess the MR analysis results. Pleiotropy was assessed using varying methodologies dependent on the number of SNPs present. The MR Egger intercept was utilized for detecting pleiotropy in cases where more than two SNPs were included. In comparison, the MR-PRESSO global test was employed when more than four SNPs were accessible (53). The outcomes of the sensitivity analysis were graphically represented through scatter plots and funnel plots. Furthermore, a leave-one-out analysis was performed on the IVs included in the study to assess their influence on the results of the MR analysis.

### 2.5 Meta-analysis

A meta-analysis was conducted to consolidate and comprehensively evaluate the collective causal effects of diverse trace element statuses on upper gastrointestinal ulcers, building upon initial findings that yielded significant results. This method enabled the synthesis of data and assessment of the cumulative influence of various trace element statuses on the pathogenesis of these ulcers.

## 3 Results

### 3.1 Bidirectional causal estimation of various trace element statuses on upper gastrointestinal ulcers in the initial analysis

**Figure 1:**
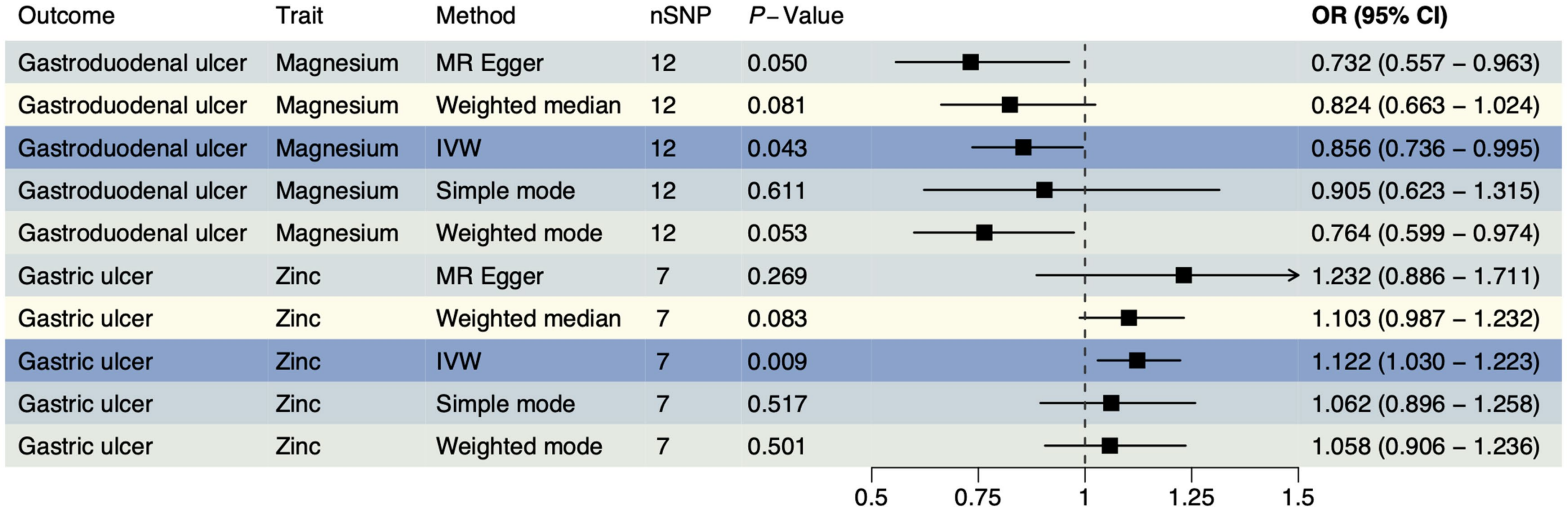
The forest plot shows the causal effect estimates from magnesium and zinc to upper gastrointestinal ulcerative diseases in the initial analysis. CI, confidence interval; IVW, inverse-variance weighted; nSNP, total number of the SNPs; OR, odds ratio.

In accordance with MR guidelines, we have identified SNPs that are closely linked to various trace element statuses. Following the exclusion of confounding factors and selection based on F-statistics, we have identified a total of 36 SNPs associated with cobalamin (VB_12_), magnesium, and pyridoxine; 24 SNPs with related to iron; 21 SNPs each associated with calcium ionized, retinol, VD, and zinc; 18 SNPs each associated with copper and folate; and 15 SNPs each associated with phosphate and selenium (Supplementary Table S1). An IVW method was used as a primary method, along with four MR methods as supplementary. According to the FinnGen database, magnesium had a reverse causal effect on gastroduodenal ulcers (Figure 1), which was statistically significant (OR < 1, P_IVW_ = 0.043), suggesting that magnesium may reduce the risk of gastroduodenal ulcers. A positive causal effect was found between zinc and gastric ulcers, also in the FinnGen database, indicating that zinc may increase the risk of gastric ulcers (Figure 1, OR > 1, P_IVW_ = 0.009), which implies that zinc may increase the risk of gastric ulcers. The association between other trace elements and upper gastrointestinal ulcers was not statistically significant (*p* > 0.05), indicating a weaker association. A detailed summary of the MR analysis results is included in Supplementary Table S2. The reverse MR analysis was performed with three types of upper gastrointestinal ulcers as exposures. Based on quality control of the IVs, we conducted MR analyses using 252 SNPs across 11 different types of trace element statuses. The SNPs are detailed in Supplementary Table S3. There were no notable causal relationships between upper gastrointestinal ulcers and various trace element statuses according to the reverse MR analysis (Supplementary Table S4), suggesting that upper gastrointestinal ulcers are unlikely to affect the metabolic processes of trace elements.

### 3.2 Bidirectional causal estimation of various trace element statuses on upper gastrointestinal ulcers in the validation analysis

**Figure 2:**
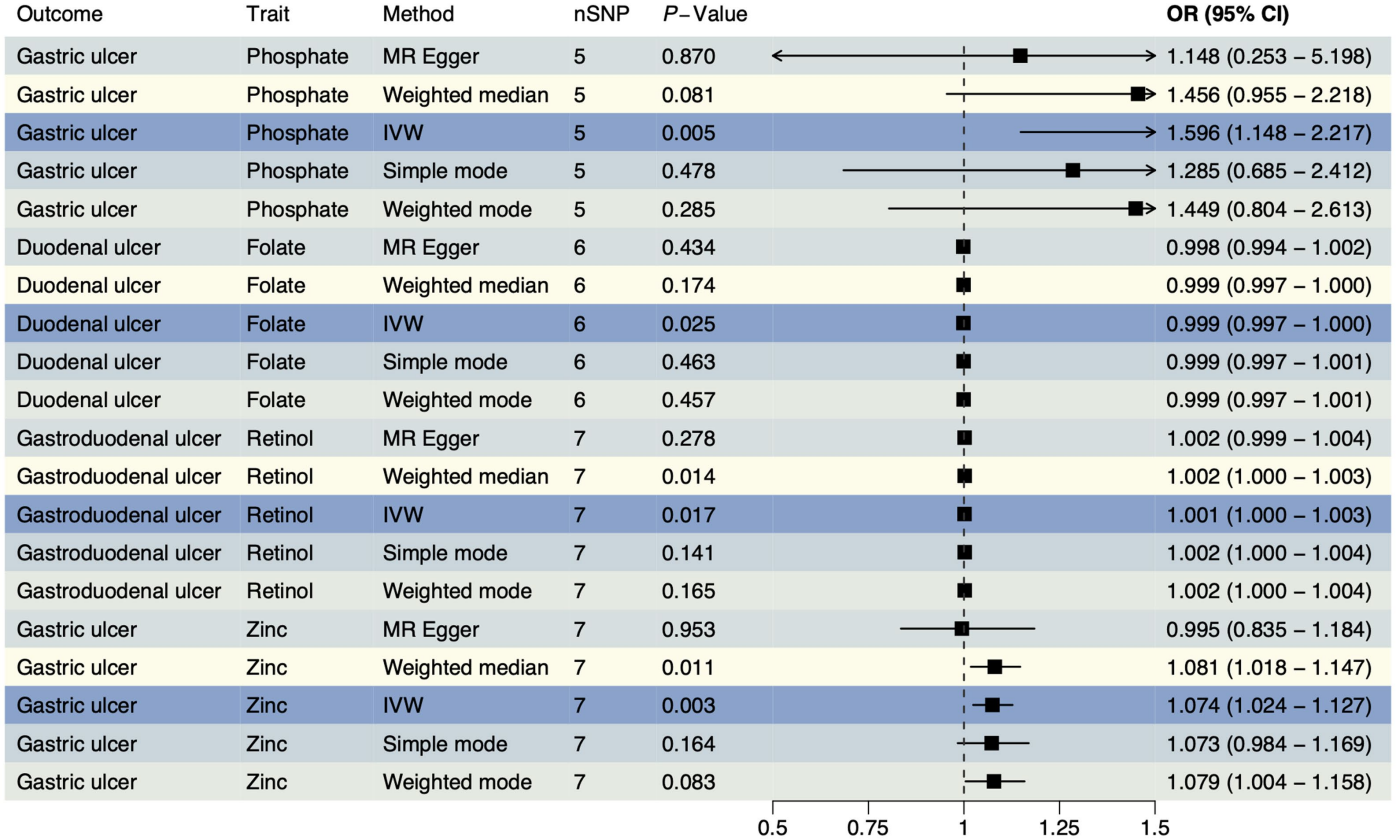
The forest plot shows the causal effect estimates from phosphate, folate, retinol, and zinc to upper gastrointestinal ulcerative diseases in the validation analysis. CI, confidence interval; IVW, inverse-variance weighted; nSNP, total number of the SNPs;OR, odds ratio.

To confirm the causal effects of various trace element statuses on upper gastrointestinal ulcers, we adapted the outcome data from the OpenGWAS database to GWAS summary data from a European cohort and conducted the MR analysis again. In this analysis, we obtained 36 SNPs each for magnesium and pyridoxine, 34 for cobalamin (VB_12_), 23 for iron, 21 each for VD and zinc, 20 each for ionized calcium and retinol, 18 for folate, and 17 for copper, with 15 each for phosphate and selenium (Supplementary Table S5). The validation analysis results (Figure 2) showed significant causal effects of folate on duodenal ulcers with a reverse causal effect (OR < 1, P_IVW_ = 0.025) and retinol on gastroduodenal ulcers with a positive causal effect (OR > 1, P_IVW_ = 0.017). However, both phosphate (OR > 1, P_IVW_ = 0.005) and zinc (OR > 1, P_IVW_ = 0.003) exhibited a positive causal effect on gastric ulcers. Details of all results from this analytical direction are listed in Supplementary Table S6. For reverse MR analysis, we used three types of upper gastrointestinal ulcers as exposures, and after quality control of IVs, we obtained 393 SNPs for MR analysis with 11 different types of trace element statuses as outcomes, which are detailed in Supplementary Table S7. Reverse MR analysis showed no significant causal relationships (Supplementary Table S8). The evidence from validation analysis highlighted that higher zinc levels may increase the risk of gastric ulcers. Moreover, these findings partially reveal how magnesium, folate, retinol, and phosphate influence the development of upper gastrointestinal ulcers, reflecting the complex mechanisms by which trace elements regulate gastrointestinal health. This provides a critical perspective on how nutrients may regulate health through physiological and pathological processes.

### 3.3 Sensitivity analysis

**Figure 3:**
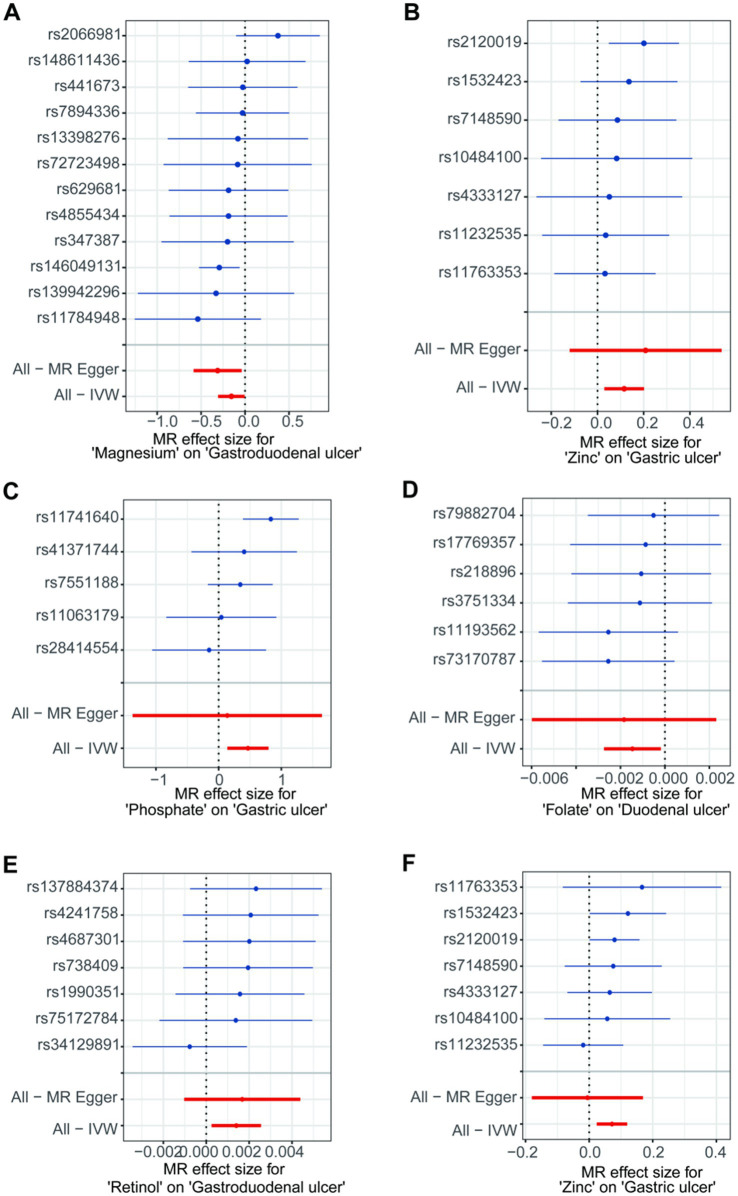
The forest plots for single nucleotide polymorphism (SNP) in the directions of different trace element states to upper gastrointestinal ulcerative diseases. (A,B) For initial analysis. (C–F) For validation analysis.

In the initial analysis, SNPs showed that rs146049131 in the direction between magnesium and gastroduodenal ulcer exhibited a statistically significant inverse causal relationship with the outcomes, while other single SNPs did not show significant causal effects (Figure 3A). However, the overall causal effect estimates using the IVW method were significant and consistent with the MR analysis results (Figure 3A). From the direction between zinc and gastric ulcer, rs2120019 showed a significant positive causal relationship (Figure 3B), while other SNPs did not exhibit significance, yet the overall causal effect estimate remained significantly positive, aligning with MR analysis results, including similar outcomes in the validation analysis (Figure 3F). Additionally, in the validation analysis, rs11741640 from the phosphate and gastric ulcer analysis direction showed a significant positive causal effect (Figure 3C), while individual SNPs in the folate and duodenal ulcer (Figure 3D), and retinol and gastroduodenal ulcer directions (Figure 3E) did not exhibit significant effects.

**Figure 4:**
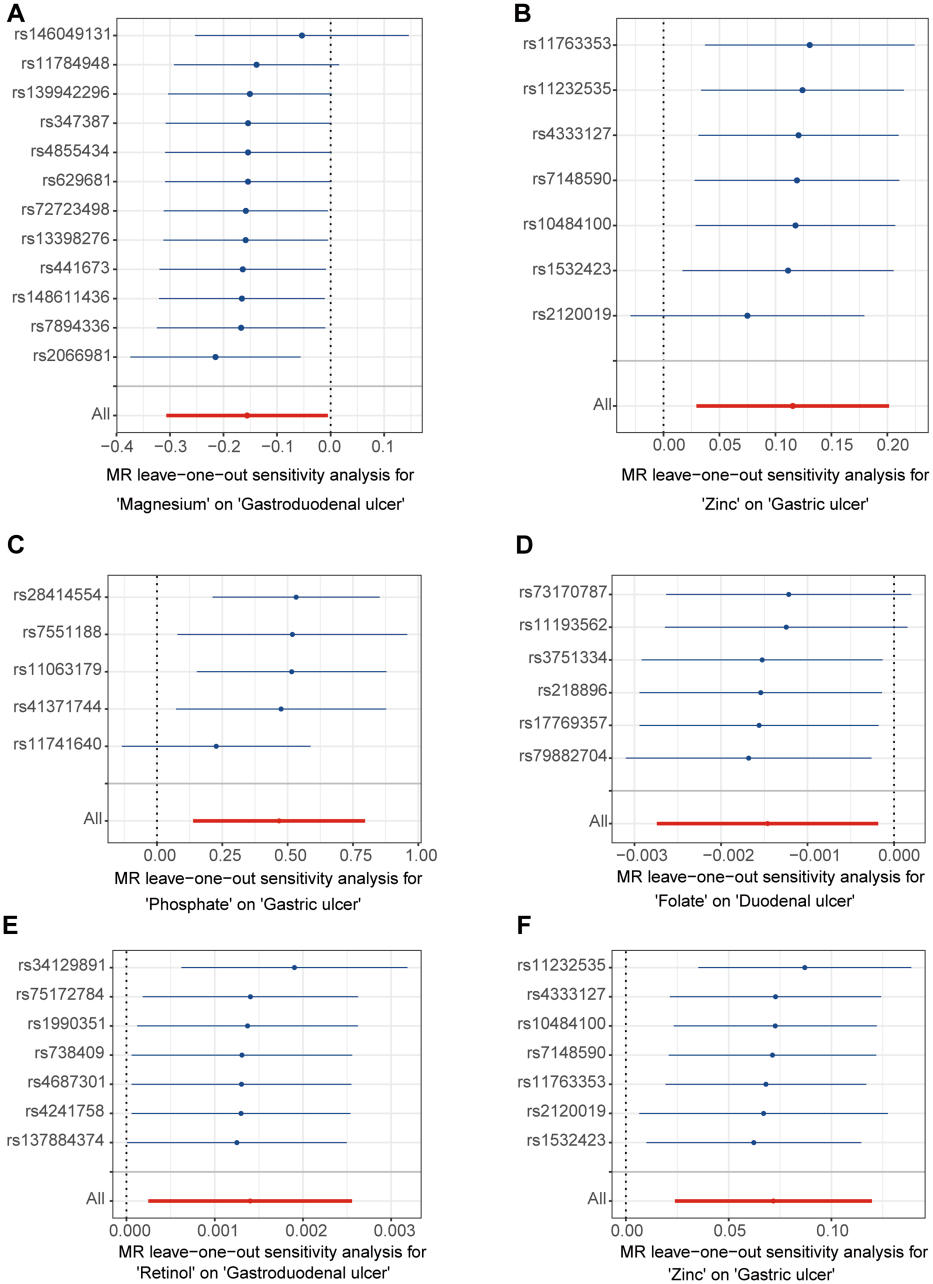
The forest plots for leave-one-out analysis in the directions of different trace element states to upper gastrointestinal ulcerative diseases. (A,B) For initial analysis. (C–F) For validation analysis.

**Figure 5:**
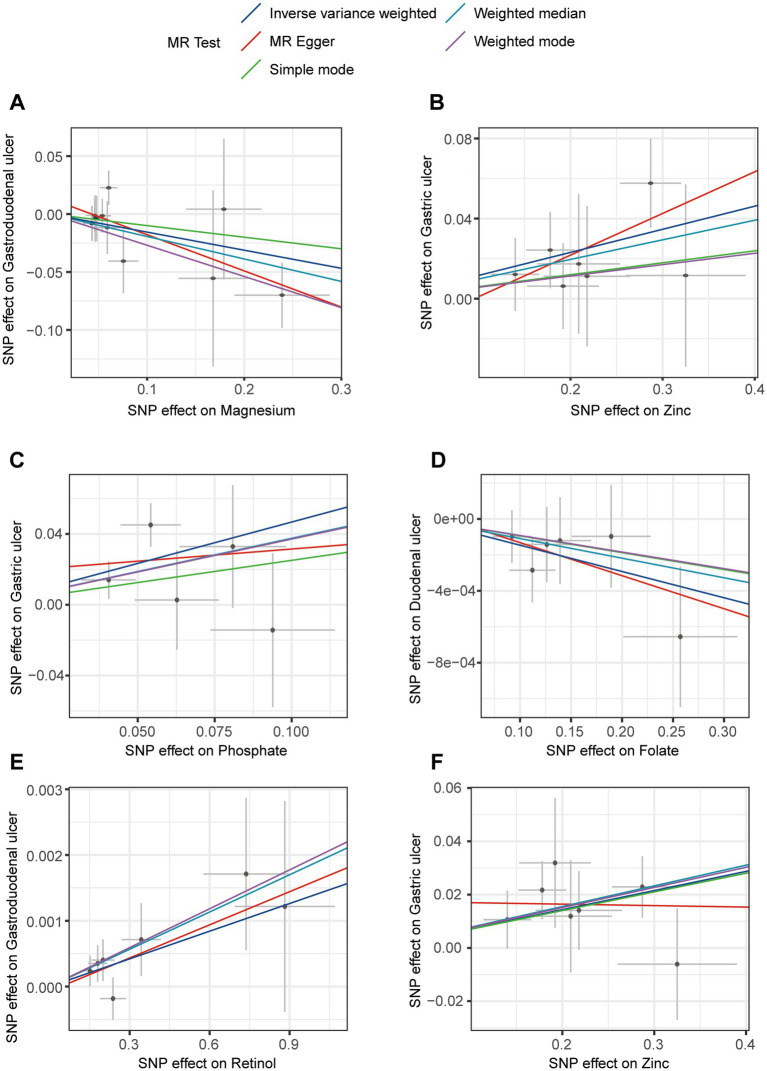
Scatter plots for sensitivity analysis in the directions of different trace element states to upper gastrointestinal ulcerative diseases. (A,B) For initial analysis. (C–F) For validation analysis.

**Figure 6:**
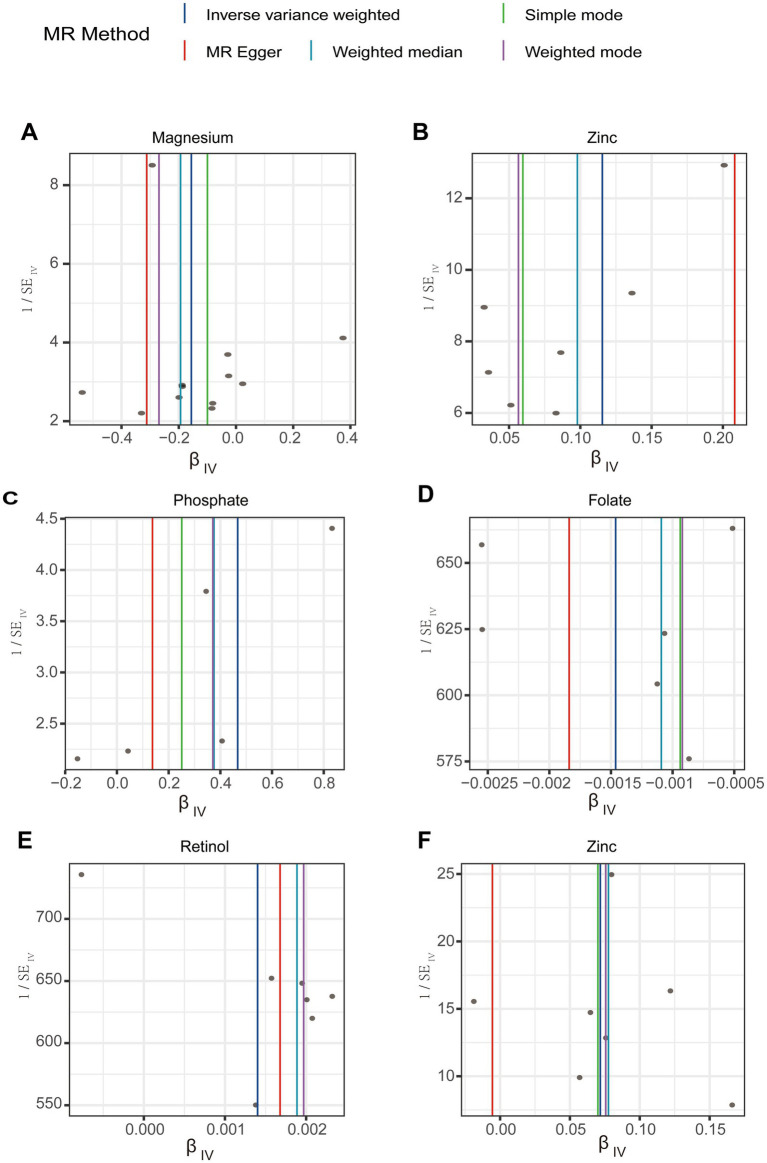
Funnel plots for sensitivity analysis in the directions of different trace element states to upper gastrointestinal ulcerative diseases. (A,B) For initial analysis. (C–F) For validation analysis.

A leave-one-out analysis was conducted to assess the impact of excluding individual SNPs on the causal effect trends of the remaining SNPs. In the initial analysis (Figures 4A,B), when excluding specific SNPs associated with magnesium, the remaining SNPs did not show statistically significant effects on gastroduodenal ulcers (Figure 4A), but excluding others revealed significant inverse causal effects (Figure 4A). With zinc, only after excluding rs2120019 did the remaining SNPs show no statistically significant impact on gastric ulcers (Figure 4B), yet in the validation analysis, excluding rs2120019 still resulted in statistically significant effects (Figure 4F), highlighting the critical role of rs2120019 in the development of gastric ulcers. This SNP may regulate gene expression or participate in critical metabolic pathways, thus affecting gastric mucosal protection or acid environment regulation. In the validation analysis, excluding specific SNPs from the phosphate and gastric ulcer direction (Figure 4C), the folate and duodenal ulcer direction (Figure 4D), and the retinol and gastroduodenal ulcer direction (Figure 4E) resulted in statistically significant effects in their respective directions. No evident pleiotropy or heterogeneity was observed across the analysis directions (Supplementary Table S9). Scatter plots (Figure 5) and funnel plots (Figure 6) also did not show any significant SNP distribution abnormalities in significant analysis directions.

### 3.4 Meta-analysis

**Figure 7:**
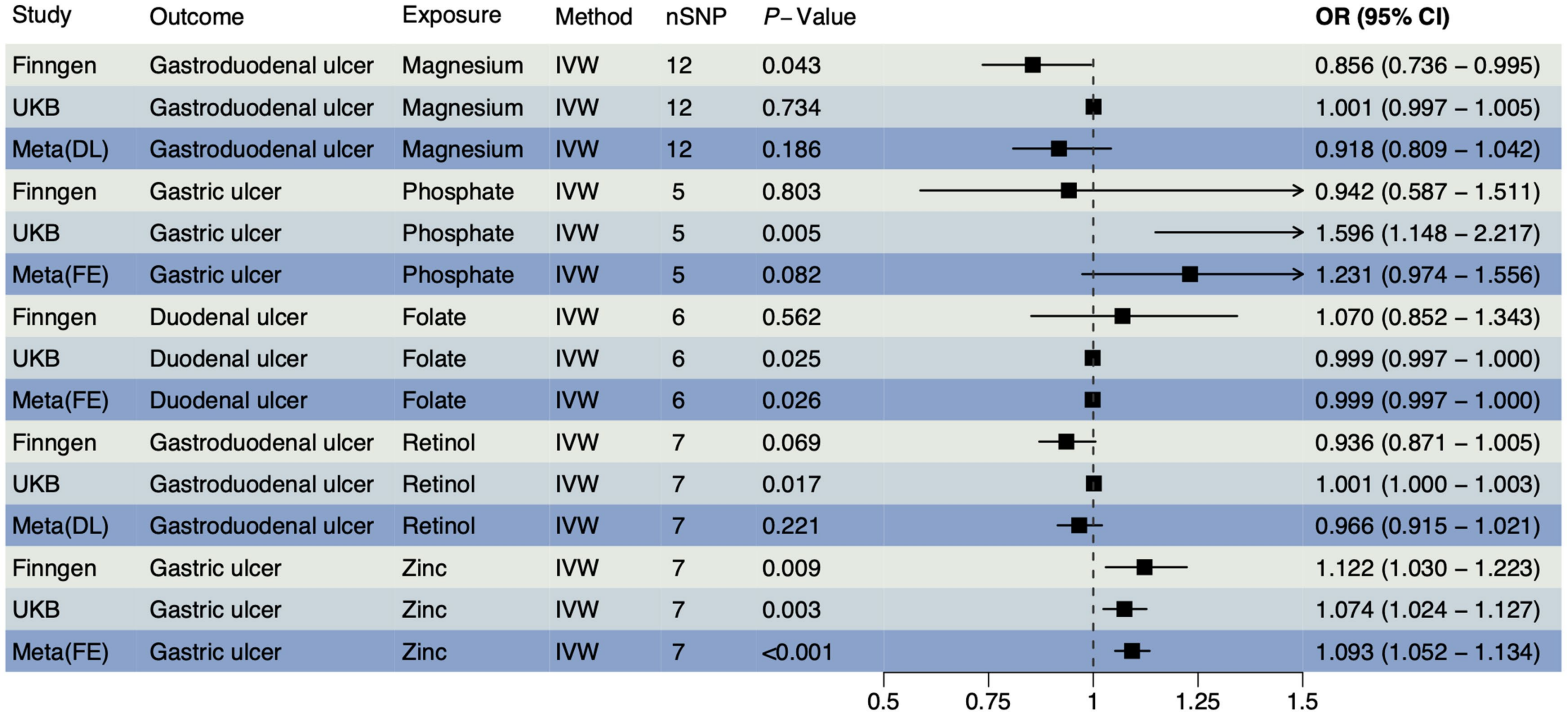
Meta-analysis for different Mendelian randomization (MR) results.

Based on the results of the initial and validation analyses, we confirmed that magnesium, zinc, phosphate, folate, and retinol have statistically significant causal effects on upper gastrointestinal ulcers across different databases. Meta-analyses of these results revealed that only the causal relationship between zinc and gastric ulcers was statistically significant across various data sources (Figure 7), reaffirming zinc’s significant causal impact on gastric ulcers. Detailed meta-analysis data are provided in Supplementary Table S10. These results suggest that further exploration of zinc’s specific role in gastric mucosal defense and repair mechanisms is necessary, including its potential regulatory effects on anti-inflammatory and antioxidant pathways, as well as determining its optimal supplementation levels for the prevention and treatment of gastric ulcers. This research study’s direction not only helps us better understand zinc’s physiological and pathological roles but also may guide the development of new treatment strategies to alleviate clinical symptoms of gastric ulcers and improve patient quality of life.

## 4 Discussion

This study employed a two-sample MR approach to explore the bidirectional causal relationships between various trace element statuses and upper gastrointestinal ulcers. Our findings suggest that elevated levels of zinc and phosphate may increase the risk of gastric ulcers, whereas magnesium and folate offer potential protective effects against gastroduodenal ulcers. The meta-analysis conducted within this research further confirmed a positive causal relationship between zinc levels and gastric ulcers, underscoring the importance of maintaining balanced zinc levels to mitigate the risk of gastric ulcers. The MR methodology leverages genetic variants as IVs to estimate the causal effects of exposures (such as specific trace element statuses) on outcomes (such as ulcerative diseases) (54, 55). This analytical approach helps eliminate potential confounders, including lifestyle factors and unmeasured biomarkers, thus providing more reliable causal inferences (56, 57). Moreover, by integrating and analyzing data from various studies, the meta-analysis supports a causal relationship between trace element statuses and upper gastrointestinal ulcerative diseases. This method enhances the statistical power and breadth of the research, enabling a more confident assertion of the role of trace elements in gastrointestinal diseases. These findings not only enrich our understanding of the role of trace elements in digestive system diseases but also provide crucial scientific evidence for future prevention strategies and therapeutic interventions. Particularly in clinical settings, managing dietary trace element content could be an effective prevention and treatment approach for patients with gastric and duodenal ulcers. However, excessive zinc might exacerbate mucosal damage, thereby promoting ulcer formation. Therefore, zinc supplementation should be carefully considered to avoid excessive intake and effectively manage the risk of gastric ulcers. For patients with existing gastric ulcers, adjusting diet and potential zinc supplementation to achieve optimal zinc levels could help alleviate inflammation and promote the healing of the gastric mucosa. Thus, it is essential to balance trace element intake carefully in practical applications.

The main benefit of the MR method is its ability to address confounders and reverse causality issues in observational studies (56, 58). Selecting appropriate IVs is essential for reliable MR analysis. In this study, we chose genetic variants strongly linked to trace element statuses from GWAS databases to meet MR analytical requirements. F-statistic calculations were conducted to evaluate the efficacy of the IVs in facilitating reliable causal inference through genetic variants (39, 59). Initially, outcome populations that were not associated with the exposure group were selected, and subsequently, validation analysis was performed utilizing the extensive GWAS data from the same database. A meta-analysis was conducted to combine results and assess overall causal effects from various data sources. Sensitivity analysis was also performed to verify the robustness of the results. MR-Egger regression and Cochran’s Q statistic were used to assess bias and heterogeneity in the results, and no significant findings have been observed. Overall, the study rigorously followed established guidelines for MR methodology, IV selection, quality control measures, and sensitivity analysis, yielding comprehensive insights into the association between various trace element statuses and upper gastrointestinal ulcers. These results enhance our comprehension of the causal links between trace elements and upper gastrointestinal ulcers and offer a robust scientific basis for the development of future prevention and treatment approaches.

Even though MR was used to establish causal relationships between certain trace element statuses and gastric ulcers, further investigations are needed to discover the specific biological mechanisms involved. The trace elements studied play crucial biological roles in the human body, including participation in cellular metabolic processes, enzyme activity regulation, and protection of cells from oxidative damage as antioxidants (60–64). The mechanisms by which zinc and phosphate increase the risk of gastric ulcers may involve multiple biological pathways, including direct damage to the gastric mucosa and modulation of gastric acid secretion. For instance, zinc is known to regulate gastric acid secretion, potentially by activating the H^+^/K^+^ ATPase in gastric wall cells, leading to increased gastric acid production (65, 66). Excessive gastric acid can damage the gastric mucosa, increasing the risk of ulcer formation. Moreover, phosphate, an essential molecule in cellular metabolism, may accumulate in gastric mucosal cells, disrupting intracellular electrolyte balance and pH levels (67). This disruption can lead to cellular dysfunction and death, thereby promoting ulcer formation. This effect might occur by impacting intracellular signaling pathways, such as MAPK and NF-kB pathways, which play critical roles in regulating inflammatory responses and cell apoptosis (68–70). Conversely, the protective effects of magnesium and folate may operate through different mechanisms. Magnesium, essential for the activity of many enzymes, is crucial for DNA synthesis, protein production, and cellular energy metabolism (71–74). Its anti-inflammatory properties might reduce gastric mucosal inflammation by decreasing the production of inflammatory mediators and regulating immune cell function. Folate plays a significant role in cell repair and metabolism, promoting the healing of damaged mucosa, potentially by enhancing the proliferation of damaged cells and inhibiting the release of inflammatory mediators (75–78). The actions of these trace elements are not limited to single biological pathways; they may operate within more complex networks involving oxidative stress responses, immune regulation, and interactions with other nutrients. For example, zinc and magnesium levels might influence each other’s absorption and utilization (25, 79), and they may also interact with the metabolism of VD and vitamin B, collectively regulating the defense mechanisms and repair processes of the gastric mucosa.

Even though MR studies have provided a fascinating insight into the potential causal relationships between trace element status and upper gastrointestinal ulcerative diseases, genetic variant studies still face many challenges. First, upper gastrointestinal ulcers are complex diseases influenced by multiple genes and factors, involving numerous genetic variants with relatively small effects, increasing the research study’s complexity and sample size requirements (80). Although some genetic variants are definitively associated with upper gastrointestinal ulcers, whether these variants directly influence the risk of upper gastrointestinal ulcers requires further research and validation. Moreover, while genetic variants implicated in MR studies are commonly perceived as stable and impervious to external influences, it is important to acknowledge that the interactions with environmental factors, individual lifestyle choices, and other genetic determinants may modulate their biological effects. Such interactions can alter the significance of MR analysis findings by potentially modifying the effects of genetic variants on disease susceptibility (81). Several directions must be explored in future research to better understand the causal relationships and promote effective prevention and treatment strategies. First, experiments at both the animal model and cellular levels are imperative to authenticate and elucidate the precise functions of trace elements in the pathogenesis of gastric ulcers. These experiments allow for the direct examination of the influence of trace element levels on the viability, programmed cell death, and inflammatory reactions of gastric mucosal cells, thereby establishing a basis for subsequent mechanistic investigations. Second, comprehensive clinical trials involving a diverse range of individuals, encompassing varying dietary patterns and health conditions, are indispensable for evaluating the impact of supplementing or restricting trace element statuses on the risk of gastric ulcers. These studies contribute to a more precise determination of recommended intake levels for trace elements, which serves as a foundation for clinical and public health guidance. Furthermore, investigating the interplay between trace element statuses and other medical or nutritional interventions holds significant importance. For example, considering that common NSAIDs are a significant factor in causing gastric ulcers, studying how trace element statuses affect NSAID-induced gastric mucosal damage could help develop safer and more effective drug treatment strategies. Overall, this study provides new perspectives and scientific evidence for understanding the complex relationships between trace element statuses and upper gastrointestinal ulcerative diseases. However, further exploration of specific biological mechanisms underlying these relationships remains a crucial focus for future research. Through these studies, we can not only enhance the prevention and treatment of gastric ulcers but also potentially uncover the roles of trace element statuses in other digestive system diseases, offering broader applications for promoting human health. Regarding public health strategies, this research provides a scientific basis for adjusting dietary guidelines and nutritional supplementation policies. For instance, although zinc and phosphate are essential trace elements, this study shows that their excess may be associated with an increased risk of gastric ulcers, suggesting a need for more caution in recommending daily intake. Conversely, the potential protective effects of magnesium and folate support the promotion of these elements’ supplementation in specific populations, especially in patients with a known history of ulcers. In clinical practice, these findings can guide physicians to pay more attention to patients’ trace element statuses when diagnosing and treating gastrointestinal diseases. For patients who frequently experience indigestion symptoms, doctors could recommend blood tests for trace element statuses and adjust diets or recommend specific supplements based on the results. Additionally, for patients using medications that might affect the absorption of trace elements, such as proton pump inhibitors, doctors should also monitor their trace element statuses to prevent potential mineral disorders. In summary, this study not only enhances our understanding of the role of trace elements in digestive health but also provides a scientific basis for formulating targeted prevention measures and treatment strategies. As the fields of nutritional science and genomics continue to develop, the future holds promise for developing more personalized nutritional management plans to optimize treatment outcomes and improve patients’ quality of life.

In conclusion, the two-sample MR combined with meta-analysis revealed a positive causal relationship between zinc and gastric ulcers, indicating that higher zinc levels may increase the risk of gastric ulcers. Additionally, this method also demonstrated potential causal relationships between other trace elements such as magnesium, phosphate, and folate and upper gastrointestinal ulcerative diseases. These findings support dietary recommendations for trace element intake and emphasize the importance of considering trace element balance in clinical and public health practices, particularly in preventing and treating gastric and duodenal ulcers. Future research should include diverse populations and environments to confirm these findings and provide accurate data for global nutrition and public health efforts.

## Data Availability

Publicly available datasets were analyzed in this study. This data can be found at: Open GWAS project (https://gwas.mrcieu.ac.uk/), and the Finngen database (R10 version) (https://r10.risteys.finngen.fi/).

## Footnotes

^1^ https://gwas.mrcieu.ac.uk/

^2^ https://ldlink.nih.gov/?tab=ldtrait
